# Assessment of job satisfaction and work ability in a confectionery factory : A Cross-Sectional Study

**DOI:** 10.1192/j.eurpsy.2024.1242

**Published:** 2024-08-27

**Authors:** H. Daoud, I. Sellami, C. Ben Chabene, A. Haddar, M. A. Ghrab, K. Jmal Hammami, M. Hajjaji, M. L. Masmoudi

**Affiliations:** ^1^Occupational medicine, Hedi Chaker Hospital; ^2^Sfax University, Sfax; ^3^Occupational medicine, Tunis, Tunisia

## Abstract

**Introduction:**

Job satisfaction is a fundamental pillar of the modern workplace. Recognizing the significance of job satisfaction and actively promoting it has become a strategic imperative in today’s work environment.

**Objectives:**

The present study aimed to assess job satisfaction and work ability among workers in a confectionery factory.

**Methods:**

A cross-sectional study conducted in a Sfax confectionery factory included 200 workers. Data were collected between December 2022 and July 2023 using a pre-established questionnaire. This questionnaire encompassed an evaluation of socio-demographic and professional data, measuring the degree of professional satisfaction and the level of work ability using a visual analogue scale ranging from 0 to 10.

**Results:**

The gender ratio was 0.64. The mean age was 33.2 ± 8.8 years. Among our workers, 77.5% reported being satisfied with their work. The average perceived work ability score was 8.15 ± 2.087. Employees with higher levels of satisfaction were more likely to have increased work capacity (p = 0.000). Elevated job satisfaction not only boosts work capacity but can also reduce stress levels, improve overall mental well-being, and contribute to a healthier workplace environment. These factors collectively lead to higher work ability.

**Conclusions:**

These findings emphasize the importance of prioritizing employee well-being to enhance overall productivity and company success. Fostering a work environment that prioritizes job satisfaction can lead to a more productive and successful workplace.

**Disclosure of Interest:**

None Declared

